# Three-dimensional printing of patient-specific plates for the treatment of acetabular fractures involving quadrilateral plate disruption

**DOI:** 10.1186/s12891-020-03370-7

**Published:** 2020-07-10

**Authors:** Canbin Wang, Yuhui Chen, Liping Wang, Di Wang, Cheng Gu, Xuezhi Lin, Han Liu, Jiahui Chen, Xiangyuan Wen, Yuancheng Liu, Fuming Huang, Lufeng Yao, Shicai Fan, Wenhua Huang, Jianghui Dong

**Affiliations:** 1 The Third Affiliated Hospital of Southern Medical University, 183 Zhongshan Dadao West Street, Guangzhou, 510600 Guangdong China; 2 The First People’s Hospital of Foshan, Foshan, 528200 China; 3 Department of Hand Surgery, and Department of Plastic Reconstructive Surgery, Ningbo No. 6 Hospital, Ningbo, 315040 China; 4 UniSA Clinical & Health Sciences, and UniSA Cancer Research Institute, University of South Australia, Adelaide, SA 5001 Australia; 5 School of Mechanical and Automotive Engineering, South China University of Technology, Guangzhou, 510640 China; 6 School of Basic Medical Sciences, Southern Medical University, No.1023 - No.1063 Shatai South Road, Guangzhou, 510515 Guangdong China

**Keywords:** Acetabular fractures, Virtual surgical planning, Patient-specific implants, 3D printing patient-specific plates, Quadrilateral plate disruption

## Abstract

**Background:**

Complicated acetabular fractures comprise the most challenging field for orthopedists. The purpose of this study was to develop three-dimensional printed patient-specific (3DPPS) Ti-6Al-4 V plates to treat complicated acetabular fractures involving quadrilateral plate (QLP) disruption and to evaluate their efficacy.

**Methods:**

Fifty patients with acetabular fractures involving QLP disruption were selected between January 2016 and June 2017. Patients were divided into a control group (Group A, 35 patients) and an experimental group (Group B, 15 patients), and were treated by the conventional method of shaping reconstruction plates or with 3DPPS Ti-6AL-4 V plates, respectively. The efficacy of Ti-6AL-4 V plates was evaluated by blood loss, operative time, reduction quality, postoperative residual displacement, and complications.

**Results:**

The operative time and blood loss in Group B were reduced compared to Group A, and the difference was statistically significant (*P* < 0.05). There was no significant difference in reduction quality between the two groups (*P* > 0.05). Reduction quality in Group B was anatomic in 10 (66.7%), satisfactory in four (26.7%), and poor in one (6.7%). In Group A, they were anatomic in 18 (51.4%), satisfactory in 13 (37.1%), and poor in four (11.4%). Residual displacement in Group B was less than that in Group A, and the difference was statistically significant (*P* < 0.05). In Group B, one case exhibited loosening of the pubic screw postoperatively. In Group A, there was one case of wound infection, one of deep vein thrombosis (DVT) in the ipsilateral lower limb, one case of traumatic arthritis and two obturator nerve injuries.

**Conclusions:**

The 3DPPS Ti-6AL-4 V plate is a feasible, accurate and effective implant for acetabular fracture treatment.

## Background

Acetabular fracture is the most challenging injury for an orthopedist due to the complicated anatomy, complex fracture pattern and limited surgical access [1]. The goals of surgical treatment for acetabular fractures are anatomic reduction and rigid internal fixation to obtain a long-term functioning hip joint [2, 3]. For complicated acetabular fractures, disruption of the quadrilateral plate (QLP) is considered as the vital issue in surgical reduction and QLP disruption must be reduced and fixed to achieve adequate stability.

Most complex acetabular fractures are caused by high-energy injuries, which are always associated with complex fracture patterns and displacement of the QLP and the femoral head [4, 5]. The only standard treatment for complex acetabular fractures is open reduction and internal fixation (ORIF). However, the complicated anatomy, complex fracture pattern and limited surgical access result in a high level of difficulty [5]. In addition, the secure positions of screw insertions are hard to verify intra-operatively [6–8]. Consequently, achieving stable and secure fixation with a simpler surgical procedure is a key issue in acetabular fracture treatment. Recently, life-size three-dimensional (3D)-printed models have been used for surgical simulation and the pre-operative selection of internal fixation methods. The outcomes of acetabular fracture were improved when using the pre-operative 3D-printed models [9–12].

Based on these results, the current study aimed to design 3D-printed patient-specific (3DPPS) plates which could be completely adapted to the acetabular fracture sites. These 3DPPS plates were used to treat complicated acetabular fractures (involving QLP disruption) to evaluate their efficacy.

## Methods

### Patients

This study was implemented with the approval of the Ethics Committee of The Third Affiliated Hospital of Southern Medical University (approval No. 201704006). It was performed in strict accordance with the recommendations in the Guide for the Care and Use of Humans of the National Institutes of Health. The 3DPPS plates in this study have already been approved and certificated by the CFDA (Class III medical device, NO. 20163460576).

Between January 2016 and June 2017, 50 patients in The Third Affiliated Hospital of Southern Medical University Clinical Center with acetabular fractures were included retrospectively according to our inclusion and exclusion criteria. All patients were treated with ORIF at our trauma center. Preoperatively, all patients were informed that they could choose 3DPPS plates or reconstruction plates for internal fixation. They were then divided into a control group and an experimental group based on their choice. The control group (Group A) comprised 35 patients treated by the conventional method of intraoperative contouring of reconstruction plates. The experimental group (Group B) consisted of 15 patients who underwent internal fixation with a 3DPPS plate.

All patients underwent radiographic examinations including X-rays (anterior–posterior (AP) view and Judet view) and computed tomography (CT) scan (slice thickness of 1 mm) before operation. The fractures were classified according to the Judet and Letournel classification [1]. Preoperative data including age, gender, time from injury until surgery, injury mechanism and fracture classification were recorded (Table 1).

**Table 1:** Demographic and injury data

Variable	Group A(*n* = 15) mean ± SD	Group B(*n* = 35) mean ± SD	*p* value
Age (years)	46.6 ± 12.3	45.1 ± 12.6	0.604
Time of injury until surgery (days)	8.6 ± 3.0	8.1 ± 4.1	0.413
Preoperative displacement (mm)	20.41 ± 6.15	20.35 ± 6.12	0.958
Blood loss (ml)	880.0 ± 673.4	1177.1 ± 691.6	0.045
Operative time (min)	141.7 ± 52.9	170.7 ± 40.6	0.037
Postoperative residual displacement (mm)	1.51 ± 0.97	2.38 ± 1.10	0.003
Time taken to contour plates (min)		11.1 ± 3.4	
Time cost to build 3D printing model (days)	3.3 ± 0.5		
Time cost to construct 3DPPS plate (days)	3.5 ± 0.7		
Male	10	22	0.849
Female	5	13	
Mechanism of injury Falling from a height	9	22	0.797
Motor vehicle accident (MVA)	6	13	
Acetabular fracture classification			
Both-column	11	22	
Anterior Column and posterior hemitransverse	3	10	0.882
T-type	1	3	

### Inclusion and exclusion criteria

In this study, the inclusion criteria were: acute fracture (< 21 days), and unilateral acetabular fracture associated with QLP disruption. The exclusion criteria were: open fractures of the acetabulum, patients who were younger than 18 years or older than 65 years of age at the time of the injury, and fractures involving the posterior wall.

### 3D model and plate designs

**Fig. 1:**
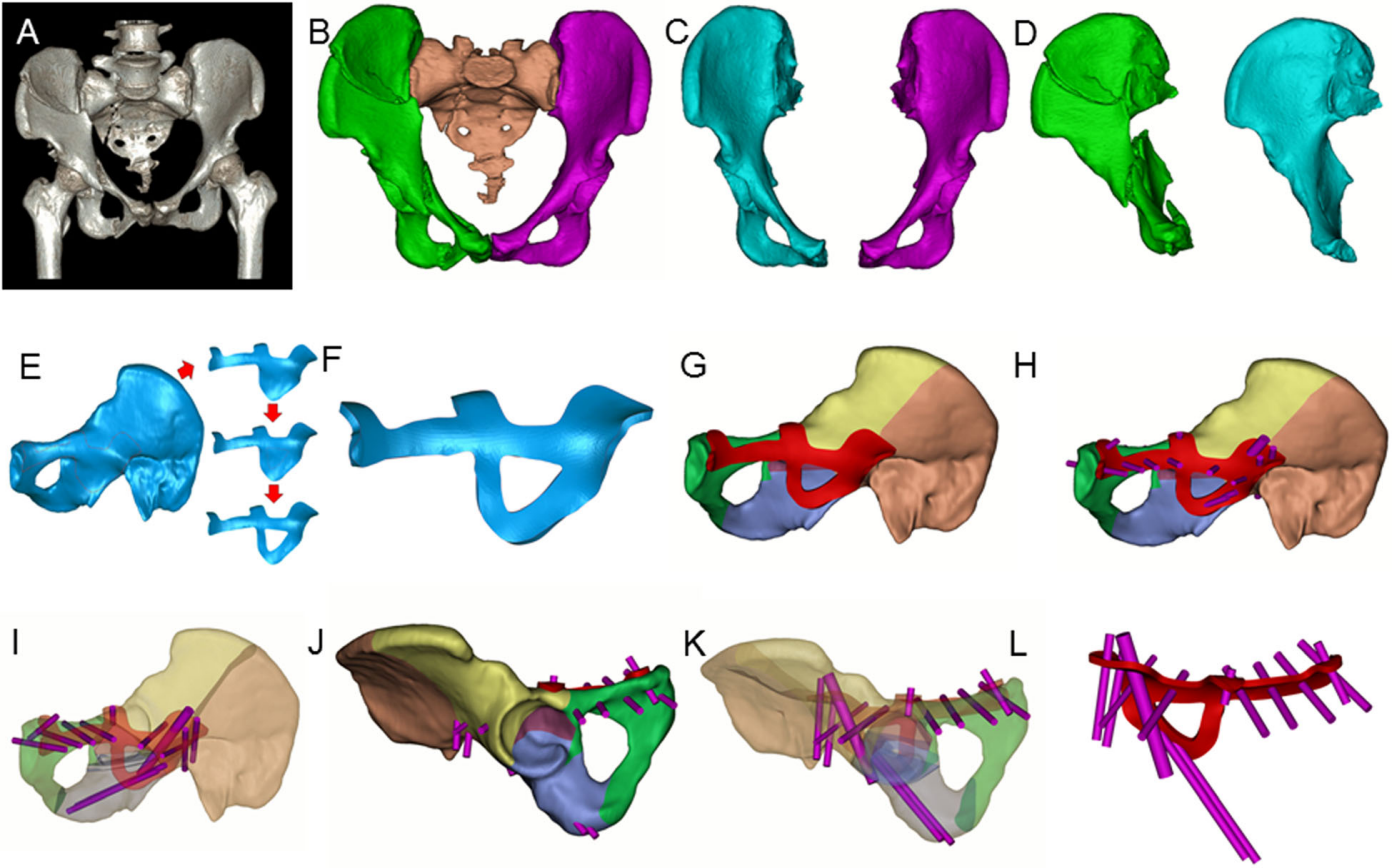
The design procedure of the 3DPPS plate**. a** 3D reconstruction of the CT scan results; **b** 3D reconstruction of the pelvis in Mimics; **c** Mirror model of the uninjured pelvis (cyan) and the model of the uninjured pelvis (purple) and **d** Model of the injured pelvis (green) and mirrored model of the uninjured pelvis (cyan). **e** The contour and position of the 3DPPS plate; **f** A virtual 3.5 mm-thick prototype plate model; **g**–**l** Virtual screw insertion in Mimics, which shows no screw penetrates into the pelvic cavity or hip joint and no overlap occurs

Radiographic data including X-rays and CT scans (Fig. 1a) were imported into Mimics 15.0 software (Materialise, Leuven, Belgium) to generate a 3D model of the pelvis. The threshold was automatically set as Bone (Min226-Max1476). Then the femur and spine were removed using the command “Edit Mask in 3D”. The mask of the pelvis was separated and reconstructed (Fig. 1b). In consideration of the bony symmetry of the pelvis, the uninjured side was mirrored as a supportive model for plate design (Fig. 1c, d).

**Fig. 2:**
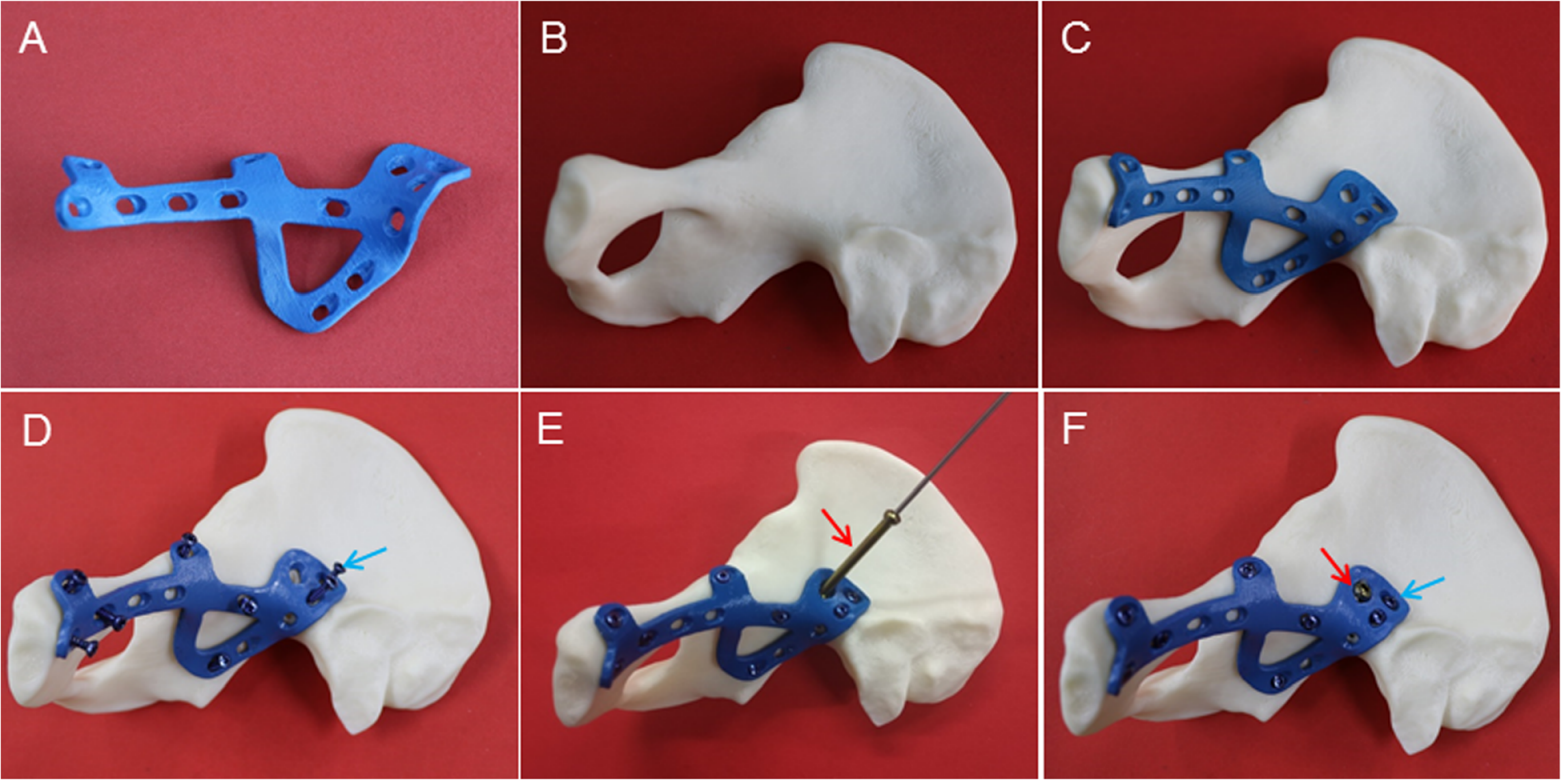
Fixation simulation on the 3D-printed acetabular model. **a** 3DPPS plate model; **b** 3D-printed acetabular model; **c** Match test of the 3DPPS plate and the 3D-printed acetabular model; **d** Simulation of all 3.5 mm screw insertions (blue arrow); **e** Simulation of 6.5 mm lag screw insertion (red arrow); **f** Fixation with the 3DPPS plate after insertion of all screws

The patient-specific acetabular plate on the mirrored pelvis was developed using Geomagic Studio 2012 (3D systems, Rock Hill, SC, USA) and Solidworks professional 2015 software (Dassault Systèmes Solidworks Corp, Waltham, MA, USA). First, the STL file of the mirrored pelvis was imported into Geomagic Studio software. Then, the model was repaired and the noise was eliminated. The contour of the plate was then designed. Subsequently, the initial designed surface was extracted using the command “Trim with curve” (Fig. 1e). Taking the fracture pattern into account, the plate was designed in the most optimal position on the mirrored pelvis to achieve stable fixation. A buttress for preventing medial displacement of the QLP was incorporated into the design of the plate, and the surface of the plate was extracted and shelled to construct a plate with thickness of 3.0–3.5 mm to ensure adequate plate strength (Fig. 1f). Finally, the models of the plate and the mirrored pelvis were imported into Mimics 15.0 and Solidworks software after boundary smoothing. Cylinders of 3.5 mm and 6.5 mm were created to simulate the insertion path of screws, and the simulations of the screw insertions included position, orientation, length and number of screws (Fig. 2g–l). The screw path must pass through the major fracture fragments and avoid penetration into the pelvic cavity or hip joint. After the insertion path of screws was determined feasible by surgeons and engineers, the corresponding screw holes were generated in the plate. The final model of the plate was obtained and saved as an STL file.

### Production process

The model of the plate was imported into the selective laser melting (SLM) 3D printer (DiMetal-100, SCUT, Guangzhou, China) and printed into a real plate using Ti-6AL-4 V powder as the raw material. The biocompatibility and mechanical properties of Ti-6AL-4 V 3DPPS plates have already been proved to be safe for clinical application, as reported in our previous study [13, 14]. Compared to the traditional plate, the 3DPPS plate shows superior biomechanical properties in biomechanical tests [12, 13]. The 3DPPS plate and the acrylonitrile butadiene styrene plastic pelvic model were matched to carry out a rehearsal of the operation before post-processing (Fig. 2a-f). Post-processing of the 3DPPS plate included heat treatment, roll casting, oil cleaning, acid pickling, polishing, anodizing and cleaning. Finally, the plate was packaged and sterilized according to the routine standards for clinical application.

### Surgical technique

**Fig. 3:**
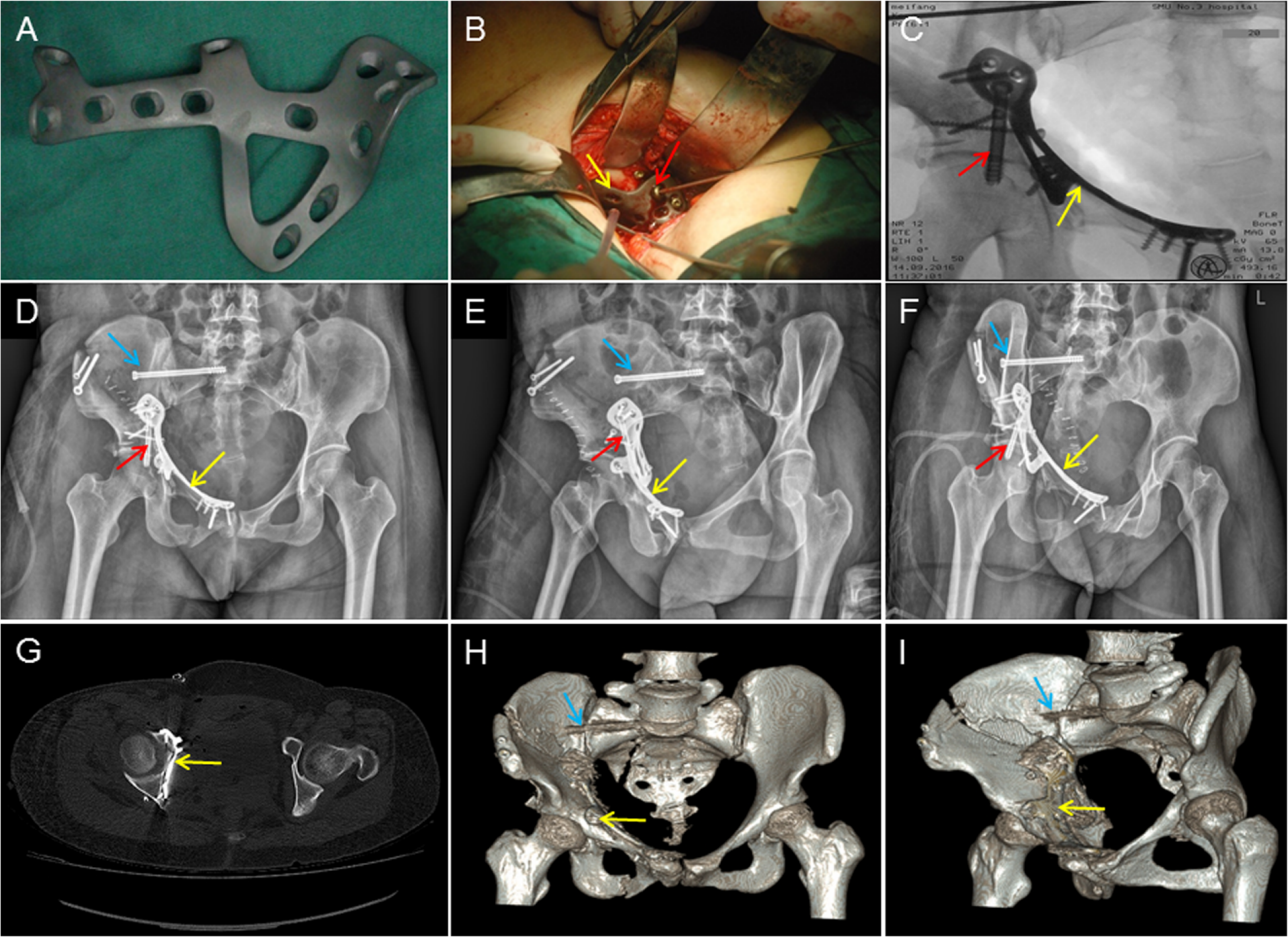
A 45-year old female who fell from a height and sustained a both-column fracture with QLP involvement, was treated with the 3DPPS plate. **a** 3DPPS plate with anodic coating; **b** Intra-operative fixation with the 3DPPS plate, yellow arrow is the 3DPPS plate, red arrow is the 6.5 mm lag screw; **c** Intra-operative radiographic data shows good reduction and fixation with the 3DPPS plate. Blue arrow is the percutaneous iliosacral screw; **d**–**e** Postoperative AP and Judet oblique view; **f** Axial images from a postoperative CT scan, demonstrating a near-anatomical adaptation of the 3DPPS plate; **h**–**i** AP view and Judet view at 3-month follow-up

The patients in Group B were informed that they would be treated with the 3DPPS plate before surgery, and all of them agreed to participate and signed the informed consent form for the 3DPPS plate. All patients received antibiotic 30 min prior to induction of general anesthesia. Patients were placed in a supine position on a radiolucent operating table and the principal surgeon stood on the opposite side to the affected hip. The single lateral-rectus abdominis approach was used to anteriorly expose the fracture site [9]. All operations were performed by one senior surgeon. The surgical technique used in the lateral-rectus abdominis approach is described in the Supplementary materials. To repair fractures, it was first necessary to reduce the medial dislocation of the femoral head by lateral traction with a Schanz pin in the lateral trochanter or by manual reduction. After the fracture of the anterior column was reduced, K-wires were used as temporary fixators to fix the anterior column in Group B if necessary. The anterior column was stabilized with a reconstruction plate in Group A. Subsequently, reduction of the QLP and posterior column was performed under direct visualization using reduction clamps or ball-spiked pushers with footplates and held with K-wires. In Group B, the 3DPPS plate was placed in the predetermined position after reduction of the fractures. By pushing the buttress design of the QLP we were able to press the quadrilateral surface back to its anatomical position. Then surgeons were able to insert all screws based on their positions in virtual pre-operative surgical planning. In Group A, an infrapectineal plate was used to buttress the posterior column and the QLP. Fixation of the posterior column was achieved by insertion of a posterior column lag screw. Fractures of the peri-sacroiliac joint and iliac crest were fixed with plates and/or lag screws. Fracture reduction and implant position were checked by fluoroscopy before wound closure. The wound was then closed in layers over drains. The drains were removed when drainage volumes were less than 50 mL per day (Fig. 3).

Postoperative X-ray and CT scans with 3D reconstruction were conducted, and prophylactic intravenous antibiotics were administered for 48 h. To prevent DVT, low-molecular weight heparin was administered daily for 2 weeks.

### Physical therapy

Physical therapy, including range of motion and muscle strength training, was initiated as early in recovery as was appropriate. Toe-touch weight-bearing was allowed for the first 8 weeks. Progressive weight bearing was allowed after radiological evidence of fracture consolidation was presented for each patient.

### Evaluation

Blood loss, operative time, postoperative residual displacement (axial image on CT), reduction quality, and complications were evaluated and statistically compared between two groups. Time for preparation of the 3D-printed model and the plate in Group B and time taken to contour the plates were also assessed. Patients received routine postoperative follow-up assessments at 4 weeks, 3 months, 6 months, 1 year and every 6 months thereafter. The evaluation criteria for reduction quality were according to the Matta scoring system (anatomic < 1 mm, satisfactory: 2–3 mm, or poor > 3 mm) [15].

### Statistical analysis

Data of age, time from injury until surgery, preoperative displacement, blood loss, operative time, and postoperative residual displacement were analyzed using the Mann–Whitney test. Categorical variables including gender and mechanism of injury were compared using the Chi-square test. Fischer’s exact test was used for comparison of fracture classification and reduction quality between the two groups. Level of significance was set at *P*-values < 0.05. All statistical analyses were conducted with SPSS 19.0 (SPSS Inc., Chicago, IL, USA).

## Results

### Demographic data

As shown in Table 1, among all preoperative data, no statistically-significant difference was exhibited between the two groups (*P* > 0.05). Meanwhile, due to the time cost of construction and transportation, the mean preparation time of the 3DPPS plates was 3.5 ± 0.7 days (range: 3–5 days) in Group B. The construction time of the 3D-printed pelvic models was 3.3 ± 0.5 days (range: 3–4 days). Simultaneously, in Group A, the mean time taken for intraoperative contouring of plates was 11.1 ± 3.4 min (range: 4–18 min).

### Operative data

When compared to Group A, the blood loss in Group B was found to be significantly lower, and the operative time of Group B was also reduced compared to Group A (Table 1). Meanwhile, compared to Group A (2.38 ± 1.10 mm; range: 0.76–4.73 mm), a significant reduction of postoperative residual displacement was exhibited in Group B (1.51 ± 0.97 mm; range: 0.63–4.32 mm). Further, according to the Matta Scoring System, in Group B 10 of the cases (66.7%) were graded as anatomic, four cases (26.7%) as satisfactory and one case (6.7%) as poor, while in Group A, 18 (51.4%) cases were graded anatomic, 13 (37.1%) satisfactory and four (11.4%) as poor, with no significant difference between the groups (Table 2).

**Table 2:** Evaluation of reduction quality based on Matta scoring system

Variable	Group A(*n* = 15)	Group B(*n* = 35)	*p* value
Anatomic (< 1 mm)	10	18	
Satisfactory(2–3 mm)	4	13	0.661
Poor (> 3 mm)	1	4	

**Fig. 4:**
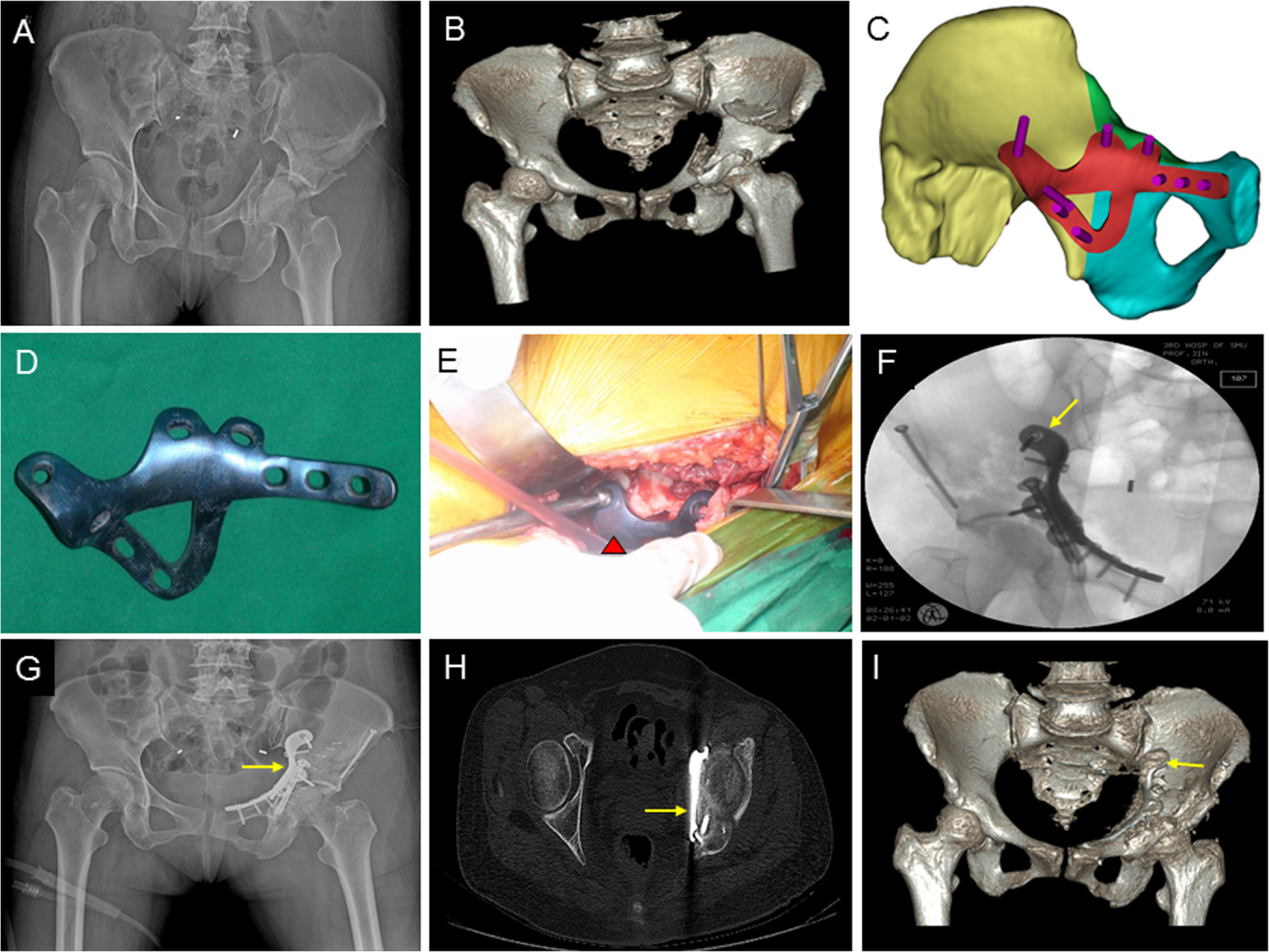
A 52-year old female, who was injured in a motor vehicle accident and sustained a both-column fracture with QLP involvement, was treated with the 3DPPS plate**. a** Preoperative AP view; **b** Preoperative 3D reconstruction of CT data; **c** Schematic design of the virtual fixation for the fractured acetabulum; **d** 3DPPS plate with anodic coating; **e** intra-operative fixation with the 3DPPS plate, red triangle indicates the 3DPPS plate; **f** Postoperative AP view; **g** Axial images from postoperative CT scan, demonstrating a near-anatomical adaptation of the 3DPPS plate **h** 3D reconstruction of postoperative CT data; **i** AP view at 6-month follow-up

All cases in this study achieved radiological evidence of fracture healing within 18 weeks. For those with follow-up of at least 1 year, none of them had loss of reduction at the final follow-up. Representative radiographic data of two cases in Group A are shown in Figs. 3 and 4.

### Complications

The complications of each group were recorded. In Group B, one patient experienced loosening of a pubic screw although the patient suffered no discomfort and radiological healing was observed at the 3-month follow-up. However, in Group A there was one wound infection, one instance of deep vein thrombosis (DVT), one case of traumatic arthritis of the hip joint and two obturator nerve injuries (Table 3). Regarding the obturator nerve injuries, both patients exhibited temporary weakness and pain of the hip adductors, but the patients recovered eventually in about 1 month without any further medical intervention. In addition, 1 patient was diagnosed with traumatic arthritis at 12 months post-operation.

**Table 3:** Complications

Variable	Group A(*n* = 15)	Group B(*n* = 35)
Loose of pubic screw	1 (6.7%)	0
Wound infection	0	1 (2.9%)
DVT	0	1 (2.9%)
Traumatic arthritis	0	1 (2.9%)
Obturator nerve injuries	0	2 (5.7%)
Total	1 (6.7%)	5 (14.3%)

## Discussion

It is difficult to achieve the ideal anatomical alignment at the interface between the plate and cortical bone with the use of a pre-bent plate for acetabular fracture, especially when this involves QLP disruption. Here, a 3DPPS plate was designed and produced to provide personalized treatment for complex acetabular fracture, enabling complete adaptation to the acetabular fracture site. These 3DPPS plates were used to treat complicated acetabular fractures and the efficacy was evaluated. The results revealed that the workflow of preparation of the 3DPPS plate was simpler and operative time and blood loss were significantly reduced.

In our study, the 3DPPS plates were designed individually according to the mirrored uninjured side of the pelvis [9, 11], they thus displayed a high level of anatomical fitting for all patients. Furthermore, our method is a simple and efficient process for plate design due to the absence of virtual separation and reduction. Conventionally, intra-operative shaping of reconstruction plates is a complicated procedure for most surgeons and even for some senior surgeons. Merema et al. [16] designed a custom-made acetabular plate for one patient by using a virtual reduced pelvis and presented a good reduction. However, virtual separation and reduction of the injured pelvis was a complicated and time-consuming procedure for complex acetabular fracture.

Maini et al. [17] reported that the mean time required to contour one 3.5 mm reconstruction plate was approximately 4.4 min. Similar to these results, an average time of approximately 11.1 ± 3.4 min (range: 4–18 min) was spent on plate shaping in Group A. Correct shaping is important, as inadequate plate shaping can result in the loss of reduction [18]. In contrast, the 3DPPS plates fitted the fracture site perfectly without the need for any manual plate bending. Moreover, the 3DPPS plates could be used as a guide to correct residual displaced fragments, and suggested an unsatisfactory reduction when the anatomically-contoured plates did not fit. Simultaneously, the position, orientation, length and number of screws had been determined during the procedure of designing the 3DPPS plate. Once the 3DPPS plate was placed on the matched fracture site, the screws could be inserted without intraoperative measurements by using the guide after drilling.

In this study, operative time and blood loss were significantly reduced (Table 1) while the rate of postoperative complications was also lower (Table 3). In acetabular fracture management, a QLP fracture is the most challenging aspect, because in addition to its deep position and high degree of comminution, a QLP fracture is always associated with medial dislocation of the femoral head which increases the difficulty of reduction [5]. Recently, various methods such as spring plates, infrapectineal plates, cerclage wires and buttress screws have been utilized for QLP fixation [19–25]; nevertheless, due to limitations caused by the low contact matching of QLP and implants, unstable and weak buttresses are still presented after QLP fixation [26]. Our design benefited from the QLP buttress design connecting the anterior column with the posterior column, so that a triangular fixation of the peri-acetabular frame was constructed to enhance the strength of fixation [27–29].

In this study, the rate of anatomical reduction in Group B was higher than that in Group A. Nevertheless, the difference in reduction quality was not statistically significant. This result indicated that the application of a 3DPPS plate is not the key element for improving reduction quality, as the use of a 3DPPS plate did not alter the surgical plans, especially for experienced surgeons [30]. The reduction skills, experience and judgment of the surgeon are still key elements for improving reduction quality. As a result, our 3D printing technology may be more helpful for young and inexperienced surgeons.

Over the past decade, 3D printing technology has shown great advantages in surgery, especially for orthopedic medicine [9, 11, 31]. However, at present the clinical applications of 3D printing technology are still limited. The primary reason for this situation is the large amount of time needed to prepare the 3D-printed object [16, 30]. In this study, in spite of establishing the optimized procedure, the time required to prepare 3D-printed models and plates was still 3.3 ± 0.5 days and 3.5 ± 0.7 days, respectively. Consequently, the time cost of preparing 3D printing still represented a risk of delayed surgery. In this study, our results proved that the 3DPPS plate is a safe and effective implant for acetabular fracture fixation and is more suitable for the treatment of complex acetabular fractures. Though some issues still limit the clinical application of such procedures, the restricted niches of 3D printing will be expanded as the technology improves and develops [32], and a larger number of patients with longer followed-up study will be observed in our further work.

## Conclusions

In conclusion, the 3DPPS Ti-6AL-4 V plate is a safe and effective implant for acetabular fracture fixation and is more suitable for the treatment of complex acetabular fractures.

## Data Availability

The authors declare no conflict of interest.

## Abbreviations

3DPPS: Three-dimensional printed patient-specific
AP: Anterior–posterior
CT: Computed tomography
DVT: Deep vein thrombosis
ORIF: Open reduction and internal fixation
QLP: Quadrilateral plate
SLM: Selective laser melting
